# Commentary on ‘Early diagnosis of serious bacterial infection in febrile infants using type I interferon signature’ by Fueri, Bellini and group

**DOI:** 10.1038/s41390-025-04474-3

**Published:** 2025-10-09

**Authors:** Scott H. Stansfield, Simon S. Craig, Marcel F. Nold

**Affiliations:** 1 Department of Paediatrics, Monash University, Melbourne, VIC Australia; 2 Monash Newborn, Monash Children’s Hospital, Melbourne, VIC Australia; 3 Ritchie Centre, Hudson Institute of Medical Research, Melbourne, VIC Australia; 4 Paediatric Emergency Department, Monash Medical Centre, Melbourne, VIC Australia

The use of biomarkers in neonatal infection and sepsis is controversial. There are strong opinions on their use, ranging from routine use to aid diagnosis and management to never—with some colleagues arguing half-jokingly that the best use for CRP is to add the blood required to measure it to the blood culture bottle. In contrast, agreement is universal that efforts aiming to reduce antibiotic overuse in N/PICUs (neonatal/paediatric intensive care units) and EDs (emergency departments) are well-justified.

Conventional markers such as CRP (C-reactive protein), PCT (procalcitonin), IL-6 (interleukin 6) and full blood count-derived measurements such as the immature-to-total neutrophil ratio (ITR) assess inflammation, which often, but not always and not reliably, accompanies infection. A perfect biomarker or biomarker score to reliably identify the source of inflammation (bacterial, viral, fungal, sterile) does not currently exist. For such a tool to be clinically useful, it would need to be a rapid, inexpensive point-of-care diagnostic with sufficient sensitivity to exclude bacterial infection (BI) in a broad range of patients so reliably and timely that the risk of not commencing empiric antibiotic therapy would become acceptably low. This in itself is an exceedingly high hurdle. Other major obstacles include the extreme variability of the status of the immune system and microbiome when they first encounter the pathogen and the dynamics of their response over time, both between different infants as well as within the same infant as she/he ages. Moreover, the diversity of the pathogens themselves as well as the initiation site of the infection and its path to becoming systemically detectable (i.e. resulting in hyper- or hypothermia, lethargy etc) add substantially to the complexity that would need to be overcome.

Therefore, studies such as that by Fueri, Bellini and group^1^ are urgently needed. In their article, the authors investigate the diagnostic utility of a type I IFN (interferon) signature in differentiating bacterial from viral infections in febrile infants aged 90 days or younger. Their prospective, single-centre cohort study recruited 47 febrile infants and 3 healthy controls, in whom they assessed the conventional biomarkers of neonatal sepsis CRP, PCT and full blood count-derived measurements such as ANC (absolute neutrophil count). In addition, the authors performed real-time quantitative PCRs to derive an IFN score from measuring six ISGs (IFN-stimulated genes), namely *IFI27*, *IFI44L*, *IFIT1*, *ISG15*, *RSAD2* and *SIGLEC1*. This score was elevated in non-BI compared to BI and healthy controls, particularly within 12 h of fever onset. The conventional markers of inflammation provided the expected differentiation between the groups, with diagnostic accuracies of 0.87 for CRP and 0.89 for PCT (ROC analyses, values are AUCs, areas-under-the-curve)—and the IFN score outperformed these markers slightly with an AUC of 0.92. The highest diagnostic accuracy was achieved by combining the IFN score with CRP or PCT (AUC 0.99 or 0.98, respectively). Furthermore, the authors highlighted that IFN scores did not vary much across different viral pathogens, and were low in patients who received antibiotics despite the absence of BI. Their conclusion is that using IFN signatures can improve diagnostic precision in early life sepsis/infection, thus potentially reducing unnecessary interventions such as antibiotic therapy, invasive procedures and hospitalisation.

The clinical dilemma of managing infants with suspected infection or sepsis lies in the nonspecific presentation of BIs and the high risk of substantial morbidity and mortality if a BI remains untreated. These circumstances lead to a high rate of precautionary (and often unnecessary) use of empiric antibiotic regimens, invasive diagnostic procedures such as lumbar punctures and urinary catheterisations, as well as hospitalisation and the associated costs to health care systems—despite the fact that up to 85% of fevers in infants younger than 90 days are viral in origin.^2^ Similarly, in N/PICU patients, empiric antibiotics are started unnecessarily in ~50% of ‘suspected sepsis’ cases,^3^ and these regimens are mistargeted in a further ~10% of cases.^4^ Blood cultures are the only widely trusted means to diagnose BIs; however, they have poor sensitivity with up to 60% false negatives in neonates,^5^ and are slow (not rarely requiring 24 h or longer to flag positive, and another ~24 h for serotyping). Consequences of the shortfall of today’s diagnostic capabilities include:

- Increasing microbial resistance to many or all available antibiotics—a major global problem, as is evident by worldwide efforts to combat it, e.g. by the Gates Foundation, the WHO and ASEAN.
- Dysbiosis, which can cause acute and chronic illness, including NEC (necrotising enterocolitis) in preterm neonates,^6^ as well as asthma and obesity in later life.^7,8^
- Delay of adequate treatment, causing short- and long-term morbidity as well as increased mortality.
- A substantial socio-economic toll; for example, ‘septic’ episodes prolong stays in neonatal and paediatric ICUs^9^ (which currently cost ~A$3,000 in NICUs^10^ and ~A$5,000 in PICUs per day in Australia) and cause co-morbidities, thus inflating N/PICU care costs, which are ~$2 billion annually in Australia in NICUs alone.^11^

Current standard biomarkers such as CRP, PCT, IL-6, ANC or ITR do not offer sufficient specificity and sensitivity to reliably guide diagnosis and management of early life infection and sepsis. Moreover, they are particularly fickle in the first hours of illness—i.e. when it matters most, because any delay in commencing antibiotic therapy increases the risk of severe morbidity and mortality if the patient has a BI. Having said this, with knowledge of the mechanisms by which these markers are induced following an infectious or inflammatory assault, and of the time courses of their responses, these markers can nonetheless be clinically useful tools. For example, once a CRP rise is first observed, the trigger most likely occurred ~12-18 h in the past; and the peak is usually reached ~24–48 h after the assault. For PCT, these times are ~6-10 h for the initial observable rise and ~18–24 h for the peak—and IL-6 is even faster, being observable ~4–6 h and peaking ~12–18 h after an infectious or inflammatory insult. Although these times can vary depending on clinical circumstances such as postnatal age, gestational age at birth, comorbidities, medications etc, with appropriate interpretation, conventional markers can make meaningful contributions to understanding inflammatory disease processes.

Aiming to obtain the best-possible diagnostic insight, it is logical to combine markers with different response-time profiles and mechanisms of induction. Fueri, Bellini and group exploited the latter aspect by measuring an IFN score, which achieved its best diagnostic accuracy when combined with CRP or PCT. Type I IFNs such as IFNɑ and IFNβ are predominantly (though not exclusively) induced by viral pathogens. Because IFNɑ and IFNβ are difficult to quantify directly, measuring genes they induce—i.e. ISGs—is a commonly employed surrogate strategy. Although the rise of ISGs is therefore slower than that of type IFNs themselves, ISGs are still among the early mediators of inflammation induced after a pathogen encounter—which renders them a good choice for a combinatory diagnostic approach. If logistical and other challenges (see below) could be overcome, novel biomarkers such as IFN scores could be considered for incorporation into routine diagnostic panels; they could also contribute to refining existing clinical prediction algorithms such as the ‘Step-by-Step’ or ‘PECARN’ tools,^12,13^ particularly in the earliest hours of illness when traditional markers are not reliably useful. Currently, both the Step-by-Step and PECARN tools classify young infants (<21 days and <28 days, respectively) as high risk, requiring a full septic workup (including lumbar puncture) and commencement of empiric antibiotics. An early and reliable method of safely identifying the majority of febrile neonates with viral illnesses may reduce unnecessary workup in this age group. Furthermore, early classification of low-risk infants may allow rapid discharge home after empiric initial therapy in the ED, instead of the traditional approach of admitting and continuing empiric antibiotics in hospital until the cultures are negative for 48 h.

As acknowledged by Fueri, Bellini and group,^1^ they report a single-centre pilot study, which is pre-clinical and has limited external validity because of the small cohort size (n = 50) and inability to perform separate training and testing to evaluate their IFN score conclusively. As such, a larger, ideally multi-site study will be required to produce clinically meaningful recommendations. The desirable broadening of applicability to N/PICUs would entail inclusion of these settings and reporting of lead symptoms (e.g. temperature instability is more common in preterm infants than fever), gestational age at birth and chronological age. On this note, it is interesting that this study demonstrates more variability in the predictive probability for IFN score in patients younger than 14 days old (Table S5 in ref.^1^).

The IFN score itself is a geometric mean of the relative mRNA abundance of six ISGs often involved in antiviral responses. Although this particular score performed well in this study, optimisation of such scores in terms of included genes/markers will be of key importance for their clinical usefulness. Moreover, mRNA-based tests are a hot topic in biomarker discovery, but are not often used to guide decisions in time-critical situations such as sepsis. Gene expression testing often requires a volume of blood (here 2.5 ml) that is high for young infants. Further, the logistics of such tests, including RNA extraction, reverse transcription and real-time PCR, are not readily available in many point-of-care settings and currently usually take too long to perform to be clinically useful for BI exclusion. Therefore, novel methods of gene expression testing to enable rapid and cost-effective profiling are needed before such tests can be employed in time-critical environments worldwide.

In summary, the shortfall of today’s capabilities to diagnose the cause of infection and inflammation results in substantial morbidity and sometimes mortality, antibiotic overuse and potentially avoidable health care costs. Novel biomarkers such as IFN scores could significantly reduce this burden in young infants and their families. Efforts to discover, validate and implement such biomarkers align with global antimicrobial stewardship goals and represent a step towards precision paediatrics, where objective molecular evidence of host-pathogen interactions plays a leading role in guiding management.

## References

[1] Fueri E. et al. Early diagnosis of serious bacterial infection in febrile infants using type I interferon signature. *Pediatr. Res.*10.1038/s41390-025-04229-0 (2025). 10.1038/s41390-025-04229-0 40537537

[2] Velasco, R. et al. Performance of febrile infant algorithms by duration of fever. *Pediatrics* **153**, e2023064342 (2024). 10.1542/peds.2023-064342 38563061

[3] Shah, B. A. & Padbury, J. F. Neonatal sepsis: an old problem with new insights. *Virulence* **5**, 170–178 (2014). 24185532 10.4161/viru.26906 PMC3916371

[4] Lin, G. L., McGinley, J. P., Drysdale, S. B. & Pollard, A. J. Epidemiology and immune pathogenesis of viral sepsis. *Front. Immunol.* **9**, 2147 (2018). 30319615 10.3389/fimmu.2018.02147 PMC6170629

[5] Buttery, J. P. Blood cultures in newborns and children: optimising an everyday test. *Arch. Dis. Child Fetal Neonatal Ed.* **87**, F25–F28 (2002). 12091285 10.1136/fn.87.1.F25 PMC1721431

[6] Cho, S. X., Berger, P. J., Nold-Petry, C. A. & Nold, M. F. The immunological landscape in necrotising enterocolitis. *Expert Rev. Mol. Med*. **18**, e12 (2016). 27341512 10.1017/erm.2016.13 PMC5001507

[7] Pascal, M. et al. Microbiome and allergic diseases. *Front. Immunol.* **9**, 1584 (2018). 30065721 10.3389/fimmu.2018.01584 PMC6056614

[8] Sun, L. et al. Insights into the role of gut microbiota in obesity: pathogenesis, mechanisms, and therapeutic perspectives. *Protein Cell* **9**, 397–403 (2018). 29725936 10.1007/s13238-018-0546-3 PMC5960470

[9] Australian Institute of Health and Welfare. *Australia’s Mothers and Babies 2016—In Brief.* Perinatal Statistics Series No. 34. Cat. no. PER 97 (AIHW, 2018).

[10] Victorian State Government, Health and Human Services. https://www.dhhs.vic.gov.au/publications/policy-and-funding-guidelines-health-and-human-services (2019).

[11] Russell, R. B. et al. Cost of hospitalization for preterm and low birth weight infants in the United States. *Pediatrics* **120**, e1–e9 (2007). 17606536 10.1542/peds.2006-2386

[12] Gomez, B. et al. Validation of the “step-by-step” approach in the management of young febrile infants. *Pediatrics* **138**, e20154381 (2016). 10.1542/peds.2015-4381 27382134

[13] Kuppermann, N. et al. A clinical prediction rule to identify febrile infants 60 days and younger at low risk for serious bacterial infections. *JAMA Pediatr.* **173**, 342–351 (2019). 30776077 10.1001/jamapediatrics.2018.5501 PMC6450281

